# Adaptive Quality of Service Control for MQTT-SN

**DOI:** 10.3390/s22228852

**Published:** 2022-11-16

**Authors:** Fabio Palmese, Alessandro E. C. Redondi, Matteo Cesana

**Affiliations:** Dipartimento di Elettronica, Informazione e Bioingegneria, Politecnico di Milano, 20133 Milan, Italy

**Keywords:** MQTT, Pub/Sub, QoS control, MQTT-SN

## Abstract

Internet of Things and wireless sensor network applications are becoming more and more popular these days, supported by new communication technologies and protocols tailored to their specific requirements. This paper focuses on improving the performance of a Wireless Sensor Network operated by the MQTT-SN protocol, one of the most popular publish/subscribe protocols for IoT applications. In particular, we propose a dynamic Quality of Service (QoS) controller for the MQTT-SN protocol, capable of evaluating the status of the underlying network in terms of end-to-end delay and packet error rate, reacting consequently by assigning the best QoS value to a node. We design and implement the QoS controller in a simulated environment based on the ns-3 network emulator, and we perform extensive experiments to prove its effectiveness compared to a non-controlled scenario. The reported results show that, by controlling the quality of service, it is possible to effectively manage the number of packets successfully received by each device and their average latency, to improve the quality of the communication of each end node.

## 1. Introduction

The Internet of Things (IoT) vision has become a reality, and the number of innovative applications based on smart and connected devices is growing considerably. Smart Cities, Industry 4.0 and connected vehicles are just a few examples of real use cases where everyday-life objects are equipped with sensors or actuators and communication capabilities and are able to either gather data from the surrounding environment and transmit it remotely or to receive commands and act accordingly. Considering the multifold areas where IoT applications can be deployed, the set of enabling technologies adopted is very heterogeneous. Especially for what concerns communication technologies, many different solutions are available [1]: at the lower layers of the protocol stack, different long-range (LoRa/LoRaWan, Sigfox, NB-IoT, LTE Cat-M) and short-range (IEEE 802.15.4/Zigbee, Bluetooth, Wi-Fi) technologies exist, each one conditioning the possibility of using IP-based protocols (e.g., 6LoWPan) at the upper layers. Similarly, at the application layer, many protocols are available, ranging from classical client/server approaches (HTTP, CoAP) to solutions based on publish/subscribe patterns, such as MQTT [2].

The latter especially has emerged in the last few years as one of the most popular application-layer protocols for IoT scenarios, due to its simplicity of use and low complexity. In MQTT, client devices connect to a central broker by means of a TCP connection and exchange data among themselves by means of publications and subscriptions over different topics. The broker is in charge of forwarding the data published to the clients interested in it, thus decoupling the process of data generation and consumption both in space and in time. This aspect, combined with the protocol simplicity at the client-side and the support for reliability and quality of service (QoS), makes MQTT well-suited for resource-constrained applications and motivates its great popularity.

To avoid the use of TCP at the transport layer and further reduce the resource requirements, a lightweight version of MQTT named MQTT-SN has been recently proposed, specifically thought for wireless sensor networks [3]. Similarly to MQTT, MQTT-SN implements quality of service control at the application layer, defining different QoS levels ranging from a simple “fire-and-forget” (not acknowledged) delivery to a more complex four-way handshake mode ensuring an “exactly once” delivery and avoiding message duplicates. QoS control is implemented in a user-centric fashion; indeed, each MQTT-SN client may select a specific QoS level for its publications or subscriptions, which in turn determine how messages are exchanged by the broker. Since MQTT-SN runs over UDP and lacks the error control provided by TCP, QoS at the application layer is fundamental in those situations when the underlying network is inherently lossy, as is the case of wireless networks, as it may constitute the only mechanism to ensure correct message delivery.

However, leaving the QoS control to each client has some drawbacks: first, the relationship between a specific MQTT-SN QoS level and the corresponding delivery performance (e.g., in terms of latency or packet loss) is not generally known to a user, as it strongly depends on the underlying network characteristics. A client may therefore request a QoS level that is too low (in case the network conditions are adverse) or too high (in the opposite case) for the desired delivery performance. At the same time, providing clients with sophisticated tools to select the optimal QoS level, such as, e.g., network bandwidth estimation [4], may be prohibitive due to the limited available resources.

To overcome such issues, this paper proposes an adaptive QoS controller that is able to tune the optimal QoS level for each node, based on specific delivery performance constraints. The contributions of this paper are the following: first, we design the QoS controller in a centralized fashion, running on top of the MQTT-SN broker; the controller gathers network statistics periodically and is able to tune the QoS of each node in order to match user-defined performance constraints in terms of end-to-end latency and packet loss rate. Second, we implement the controller in a simulated environment based on the NS-3 framework: for the task at hand, we embed into the simulator the MQTT-SN protocol and run it on top of a 802.11b IoT network composed of several clients as well as interfering nodes. Finally, we perform extensive experiments and evaluate the effectiveness of the QoS controller compared to a traditional scenario where the QoS level choice is left to the user.

The remainder of this paper is structured as it follows: Section 2 contains a review of the related literature, while Section 3 gives a background on the MQTT-SN protocol with emphasis on the QoS levels, and describes the simulation environment implemented using the NS-3 framework. The features of the QoS controller are described in Section 4, while Section 5 reports the results of the performed experimental evaluation. Finally, Section 6 concludes the work with some final remarks and future research directions.

## 2. Related Works

The topic of QoS control, management and provisioning in Internet of Things systems has been a subject of recent research attention, motivated by the massive numbers of applications that communicate with smart objects using a diverse set of communication technologies and protocols. We focus here on works explicitly targeting QoS at the application layer: more complete surveys of QoS support solutions for IoT at the different layers of the protocol stack can be found in [5,6,7].

Application layer protocols for the IoT can be roughly categorized in two classes: RESTful and Pub/Sub approaches. Protocols such as HTTP and CoAP fall within the former class, with MQTT and MQTT-SN in the latter. Recently, some effort has been put in place to make CoAP capable of Pub/Sub interactions [8], somehow indicating a preference of the IoT ecosystem for Pub/Sub solutions. Finally, there exists several less popular protocols able to work in both communication patterns, such as XMPP [9] and AMQP [10].

### 2.1. RESTful Protocols

HTTP is the most popular application-layer protocol used in the Internet and it is clearly an attractive solution for IoT applications. However, due to its synchronous client/server pattern and high header overhead, it is often left aside in favor of more lightweight options. HTTP Quality of Services relies on the underlying transport layer provided by TCP, and it is therefore not explicitly provided.

Differently, CoAP was created with the purpose of adapting the well-known HTTP functionalities to resource-constrained devices typical of IoT scenarios. CoAP is based on UDP at the transport layer: on the one hand, this choice makes it a very lightweight protocol, while on the other hand it forces to manage QoS directly at the application layer. For this reason CoAP provides a two-level QoS support for messages, which can be of unconfirmable (not acknowledged) or confirmable (acknowledged) type. In case a confirmable message is lost, it is retransmitted after a retransmission timeout (RTO), whose value is insensitive of network conditions. Indeed, the legacy version of COAP does not adapt the RTO on the basis of the network Round Trip Time (RTT). Therefore, if the RTO chosen by CoAP congestion control is below the actual RTT, CoAP will incur spurious retransmissions, which contribute to increasing network congestion. To address such shortcomings, several works proposed modification to the CoAP congestion control protocol for what concerns RTO managing [11,12].

The authors of [13] proposed a CoAP QoS support for timeliness, i.e., the end-to-end transmission delay. Clients requesting a resource to a CoAP server may express the priority (among four different levels) with which they wish to be notified by the server, which can accept or negotiate such priority. Furthermore, servers differentiate between critical and non-critical notifications, and each client may select what type of notification it is interested in. The authors show through experiments in a real wireless sensor network that the proposed modification is able to deliver on-time critical notification in the context of a e-health application.

### 2.2. Pub/Sub Protocols

In the publish/subscribe communication pattern, all communications between nodes are made available via a broker. The broker accepts messages published by devices on specific topics and forwards them to clients subscribed to those topics, ultimately controlling all aspects of communication between devices. This allows decoupling message generation and consumption in both space and time, as well as greatly decreasing the complexity of the client side communication. For these reasons, publish/subscribe protocols are of great use in the IoT ecosystem.

MQTT is the most popular pub/sub protocol currently. Based on the TCP protocol at the transport layer, MQTT allows a simple communication between devices through a central entity, named the broker, which interconnects clients sending and receiving data, named publishers and subscribers, respectively. MQTT follows the pattern of topic-based publish–subscribe communication models: subscribers can express their interest in a particular topic by issuing an appropriate request to the broker, so to receive updates every time a new message published to that topic is transmitted from a publisher. MQTT requires clients to connect to a broker before publishing messages or subscribing to topics. Each publication and subscription is coupled with a specific QoS level, chosen among three values: QoS level 0 implies fire-and-forget delivery, relying on the underlying robustness of the TCP layer; QoS level 1 refers to an “at least once” delivery, with acknowledgments required by the publisher (or to the subscriber) and retransmissions taking place in case the acknowledgment is not received; QoS 2 avoids publication duplicates with a four-way handshake among the publisher and the broker. Apart from this native QoS mechanism, several works have addressed other options for improving the quality of MQTT transmission; in [14], an adaptation framework for N-to-1 periodic communications is presented. The subscriber explicitly transmits to the publishers control messages for changing the publication rate so that the subscriber CPU utilization remains within fixed bounds. Similarly, the work in [15] addresses flow control towards subscribers. A new MQTT control message is introduced, with subscribers explicitly asking publishers to regulate their data emission. The newly proposed control message contains commands such as pause sending, unpause, send with higher rate or lower rate for each topic. Differently, the work in [16] proposes an automatic adaptation of the publication QoS level based on the estimated network RTT. The publication frequency is decreased if an increase in RTT is observed, to avoid network congestion. On the same line, the work in [17] proposes a QoS controller for MQTT in a Electric IoT scenario monitoring a power grid, among field gateways and the broker connecting them. The controller works around an optimization problem which attempts to minimize the weighted sum of packet-loss ratio and delay, which are both modeled analytically. The problem is transformed into a MAB (multi-armed bandit) problem, and solved through reinforcement learning. In detail, each field gateway estimates packet-loss values and transmission delay of each packet and calculates the reward for the current QoS configuration, in order to select the next QoS level to minimize the objective function. Some works have addressed the QoS in different terms: as an example, the work in [18] studied a distributed scenario with multiple MQTT brokers and special devices called gateways in charge of keeping track of the QoS (in terms of latency) between clients and brokers. Clients connect to gateways that select the best broker for them in order to minimize latency and perform load balancing.

Finally, very few works have addressed QoS control for the MQTT-SN protocol, the lightweight version of MQTT developed for resource-constrained scenarios. The work in [19] analyzes the end-to-end delay and delivery rate of MQTT-SN considering several system parameters, such as the publication rate, the QoS level, etc., in order to derive admission and service control policies.

The closest work to ours is [20], where authors present a QoS dynamic control aimed at maintaining a good tradeoff between end-to-end latency and packet delivery rate by automatically changing the QoS level. The method continuously monitors only the network latency and estimates the network conditions based solely on that. The QoS controller then maintains the QoS requested by a user if the estimated latency is within specific bounds, considered as normal conditions. If the network latency is above or below such specific bounds, the controller forces the minimum level of QoS. The rationale is that in case the network conditions are good, QoS 0 allows minimizing the end-to-end latency, allowing a good delivery rate; in case the network conditions are bad, higher levels of QoS (1 or 2) tend to increase the amount of traffic produced in the network (due to acks) without giving guarantees on the delivery rate (QoS 1) or end-to-end latency (QoS 2). Our work differs from the work in [20] in the following aspects: first, it is focused on the subscribers’ QoS, rather than on the publisher one; second, it accepts specific delivery performance constraints from the clients, both in terms of latency and success delivery rate: indeed, the proposed QoS controller is able to work in different operation modes optimizing either the end-to-end latency or the message hitrate; finally, we perform extensive experiments in a simulated environment allowing us to modify at will the underlying network characteristics, whereas in [20], tests were performed with a small scale testbed.

## 3. Simulating MQTT-SN

### 3.1. Protocol Overview

The MQTT protocol has emerged as the gold standard for IoT applications: as a matter of fact, all major cloud platforms (e.g., Amazon AWS, Microsoft Azure, and IBM) expose their IoT services through MQTT, and many IoT-related enterprises provide MQTT-based data collection and communication solutions.

**Figure 1:**
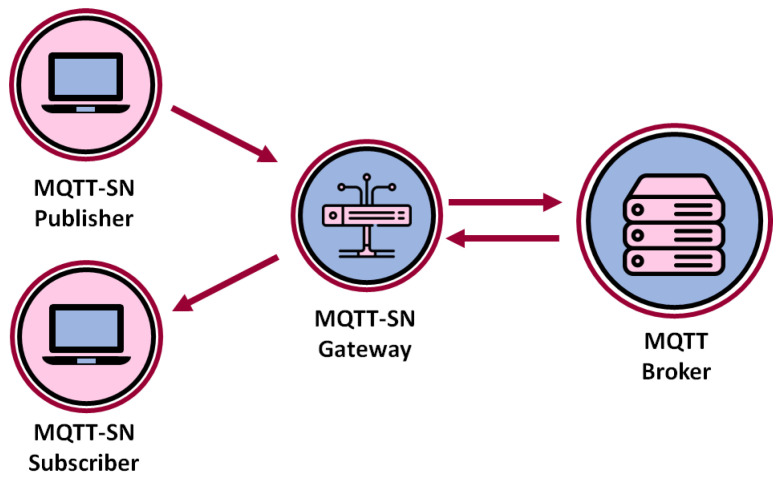
Sketch of the MQTT-SN architecture.

At the same time, MQTT requires an underlying reliable transport layer, such as TCP, that provides an ordered lossless connection capability. This may be either unavailable or too complex to handle for very simple, small footprint, and low-cost devices such as wireless sensors or in general smart devices used typically in IoT scenarios. This reason motivated the release of an adaptation of the MQTT over the UDP transport layer, with reduced size messages and additional lightweight functionalities to fit perfectly to the peculiarities of wireless sensor networks. This protocol takes the name of MQTT-SN (MQTT for Sensor Networks): a simple sketch of its architecture is reported in Figure 1. As one can see, in MQTT-SN, a Gateway device interconnects clients to a legacy MQTT broker. The role of the Gateway, which is often co-located with the broker, is to act as a translator between the two protocols, allowing the compatibility and integration of MQTT-SN with legacy MQTT applications. MQTT-SN introduces optimizations to adapt to the peculiarities of a wireless communication environment such as low bandwidth, high link failures and short message length. In particular, to cope with the latter issue, MQTT-SN allows us to refer to topics using small 2-byte topic identifiers, which are registered in the broker/gateways and mapped to legacy string-based topics. Other optimizations include the support of frequently sleeping clients through buffering, and reduced-length application layer connection procedures. In terms of QoS, MQTT-SN inherits the three MQTT quality of service levels, as described above, with an additional QoS -1 level to be used only from publishers to send messages skipping the connection phase. Clearly, due to the lack of a reliable transport layer such as TCP, QoS management at the application layer is a fundamental aspect for ensuring specific delivery performance.

Giving the clients the capability of controlling the QoS to use for each published message allows the adaptation to different and dynamic network conditions. However, selecting the best QoS is not a trivial choice for clients, which may not be aware of the underlying network characteristics. Under bad network conditions, increasing the QoS clearly leads to an increase of the reliability, but at the cost of increasing the number of messages produced and the overall delay in the communication (which may be not affordable in strict real-time scenarios). Finding the best trade-off and hence the most performing QoS to use for sending/receiving messages is crucial for improving the communication efficiency. In the particular scenario of WSNs, bringing intelligence into the constrained devices in order to automatically adapt the QoS is not always possible, due to resource limitation. For this reason, this work sets the goal of building a reactive controller to be placed in the gateway/broker with the goal of optimizing the application layer quality of service of each client analyzing simple and easy to compute performance indicators derived from the communication network.

### 3.2. Protocol Simulation

Although some implementations of MQTT-SN brokers are available (e.g., Mosquitto (https://mosquitto.org/ (accessed on 15 October 2022))), in this work we opt for a simulated approach, which allows us to better control the network characteristics as well as perform large scale experiments easily. For the task at hand, we implemented a version of the MQTT-SN protocol in the NS-3 network simulation environment (https://www.nsnam.org/ (accessed on 15 October 2022)). The framework allows us to simulate a network from the application down to physical layer, furthermore allowing us to compute and keep track of all measures needed in order to build the QoS controller (i.e., communication delay, throughput, etc.). We focus particularly on the characteristics of the MQTT-SN protocol which are related to QoS (publish–subscribe mechanisms over UDP with retransmission mechanisms), without considering technical aspects such as, e.g., connection procedures at the application layer, topic registration or last will and testament messages.

**Figure 2:**
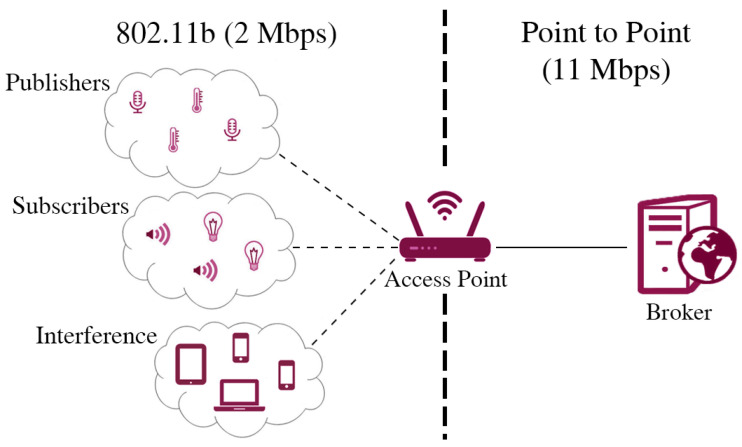
Simulator architecture.

In details, we used NS-3 to implement a classic MQTT-SN network architecture composed by a MQTT-SN broker, a MQTT-SN gateway and the various client nodes (publishers, subscribers and interference nodes). The network topology is illustrated in Figure 2 and structured as it follows:

- Client nodes connect to the broker through a IEEE 802.11b connection by means of a standard Access Point. The specific choice of 802.11b is motivated by the fact that the majority of commercially available Wi-Fi-enabled IoT devices work in the 2.4 GHz band and have no or very limited support for more recent amendments such as 802.11 ac/ax or for using higher frequency bands like the 5 GHz band. The physical layer is configured to have a bandwidth of 2 Mbit/s and a propagation delay of 2 μs. All clients use UDP protocol over IP at the transport layer.
- The MQTT-SN broker is directly connected to the access point through a point-to-point connection, with an ideal channel.

In order to simulate a real WSN use case, we implemented three different types of client sharing the same 2 Mbps channel:

- Publisher nodes: The origin of the MQTT-SN traffic; these nodes send messages to the broker at a certain rate with a specific payload to be delivered to all the subscribers (a single common topic is used)
- Subscriber nodes: The receiving end of the publish messages. After correctly receiving a packet from the publisher, the broker has to forward the publications to the subscribers that requested it (all of them in our case)
- Interference nodes: These nodes generate generic non-MQTT-SN traffic and have been introduced to create undesired traffic to mimic a real network scenario in which several devices share the wireless channel.

To simplify the scenario, we consider only one topic in the network: therefore, every message transmitted by a publisher is received by the broker and forwarded to all subscribers. For each message published by the clients (or forwarded by the broker to the subscribers), the simulator allows to control its QoS level: specific retransmission procedures, where applicable, are implemented following the best practices of the MQTT and MQTT-SN protocol specification. For QoS 0, no retransmissions are expected. For QoS 1, the standard specifies a retry timer $T_{retry}$ (in the order of seconds) and a retry counter $N_{retry}$ (between 3 and 5 retransmissions). No indications are given on how to manage the retransmission timeout in case of a transmission failure. We implemented a simple approach according to which the retransmission timer is incremented linearly by 1 s for each retransmission. The maximum number of retransmission has been set to 4. For what concerns QoS 2, the retransmission process goes on until the four way handshake specified in the standard is successful.

### 3.3. Environment Evaluation

**Figure 3:**
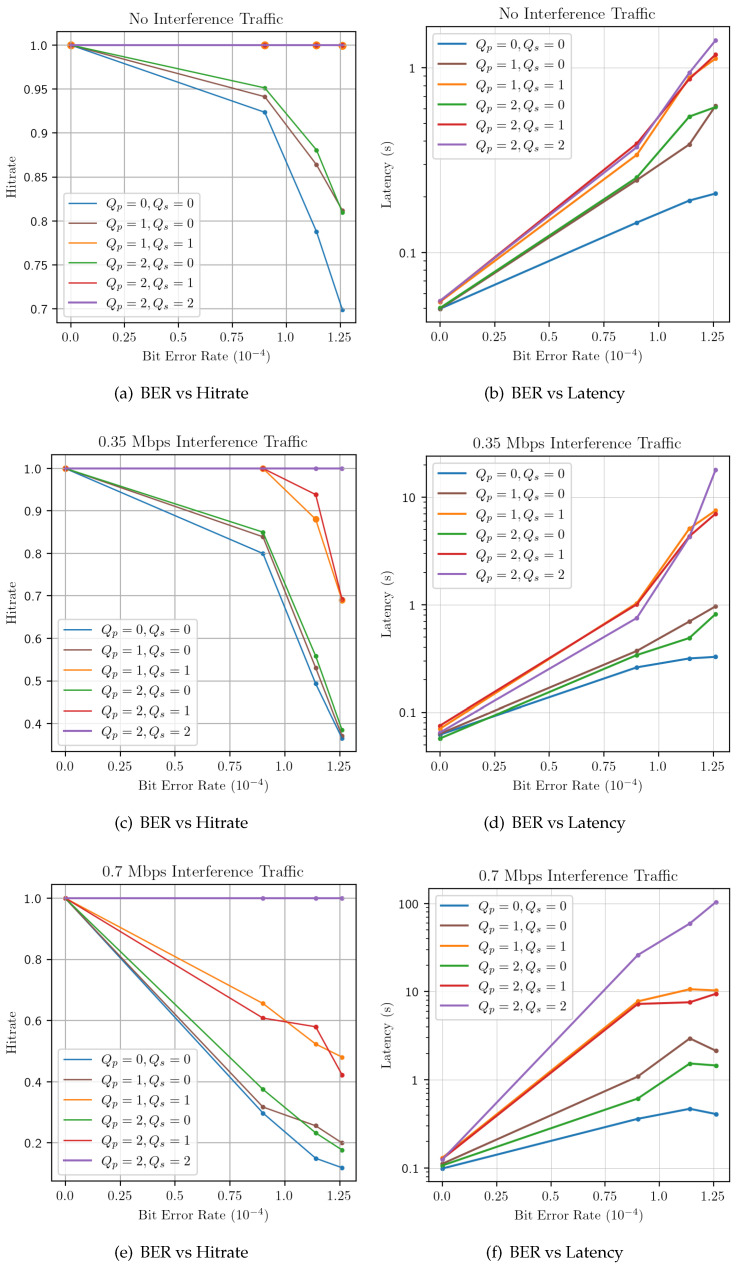
Average hitrate (**left**) and latency (**right**) for all publisher/subscriber QoS combinations under different network conditions (varying BER and Interference traffic).

The implemented simulation framework allows us to perform several tests in order to evaluate the delivery performance obtained by MQTT-SN clients in different network conditions. In particular, the following two performance metrics can easily be computed: (i) the hitrate, defined as the percentage of the published MQTT-SN messages that successfully reach the subscribers, and (ii) the end-to-end latency from a message publication to its arrival at the subscribers. We run several tests where one single client transmits 1400-bytes publish messages at a fixed rate of 1 msg/s to 10 subscribers. Each test is characterized by different underlying network conditions, in terms of the Bit Error Rate (BER) of the wireless channel as well as the amount of traffic produced by interference nodes. Moreover, in each test we controlled the QoS requested by both publishers and subscribers to evaluate its effect on the delivery performance. Let $Q_{p}$ and $Q_{s}$ be the publisher and subscriber requested QoS levels, respectively. According to the MQTT standard specification, the QoS of notifications sent by a broker to a subscriber must be the minimum of the QoS of the originally published message and the maximum QoS granted by the broker to the subscriber. Therefore, only the couples where $Q_{s}\leq Q_{p}$ have been tested. Figure 3 shows the hitrate value and latencies obtained for different QoS configurations and different underlying network conditions. Several considerations can be made by the inspection of such results:

- For what concerns the hitrate, unsurprisingly, when the network conditions are favorable (no interference traffic and low BER), the delivery performance is good regardless of the QoS level used. When the BER or the level of interference traffic increase, all QoS configurations except for $Q_{p}=2,Q_{s}=2$ suffer from a decrease in hitrate. Furthermore, it is evident how the hitrate is greatly dependent on the subscriber QoS level $Q_{s}$, rather than $Q_{p}$. Indeed, at all interference traffic levels, the curves sharing the same $Q_{s}$ (blue, brown and green for $Q_{s}$ = 0, orange and green for $Q_{s}$ = 1) follow the same trend at different BER.
- As for the end-to-end latency, similar observations can be derived. In the case of good network conditions (low BER and interference traffic), all QoS levels show in generally low latencies. Figure 3b,d,f again show a clustered behavior dominated by $Q_{s}$, with configurations sharing the same subscriber QoS having the same trend. However, the gap in performance obtained by difference configurations with the same $Q_{s}$ is evident: indeed, while at $Q_{s}=1$ the end-to-end latency is bounded by the maximum number of retransmissions set by $N_{retry}$, for $Q_{s}=2$ the latency can increase indefinitely. Indeed, while at good network condition the impact of an higher QoS level is somehow limited (although always producing higher end-to-end latencies), at high BER and interference traffic levels using QoS 2 produce latencies, which are two order of magnitude greater than in QoS 0. This is clearly due to the very high number of retransmissions performed by the broker in the attempt to deliver messages to the subscribers.

## 4. QoS Controller Implementation

**Figure 4:**
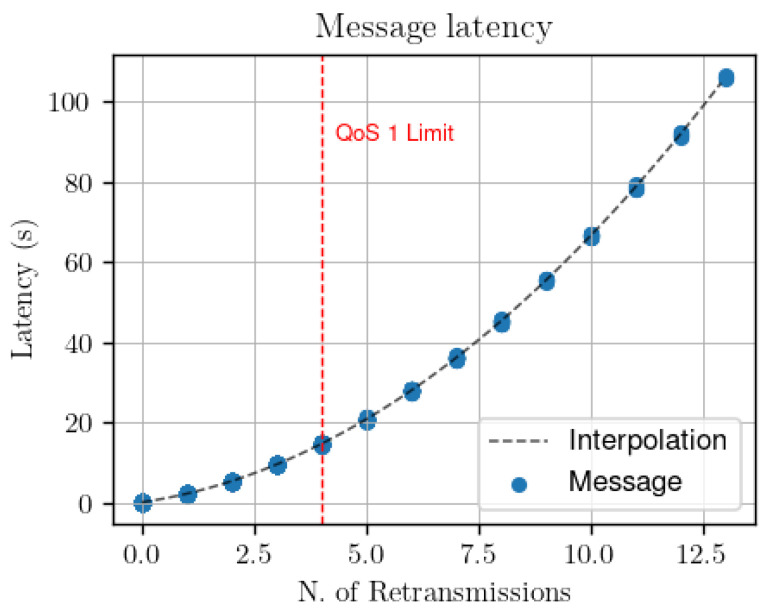
Message latency in function of the number of retransmissions using different packet error rates in the simulations.

**Figure 5:**
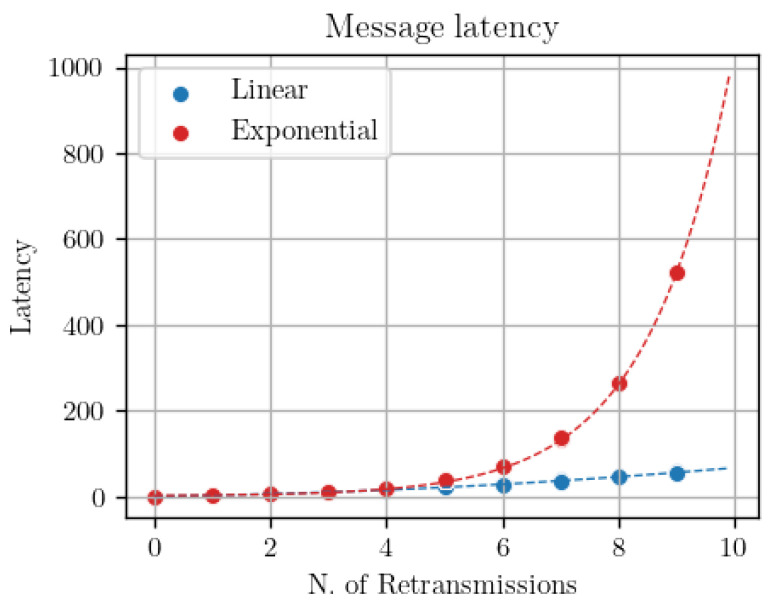
Message latency over the number of retransmission using Linear vs. exponential retransmission timeout policy.

The QoS controller has been designed to run on board the broker, leveraging its central role in the network as well as its superior computational and memory resources compared to general IoT clients. The main rationale behind the controller creation is the following: instead of asking for a particular level of QoS, IoT clients subscribing to the broker request a specific delivery performance, either in terms of hitrate or end-to-end latency. The QoS controller then selects the most appropriate QoS level to satisfy the clients’ requests. Furthermore, as emerged from the results shown in Figure 3, it is clear how the subscribers’ QoS play a lead role in determining the final delivery performance: therefore, the suggested QoS controller works only on the subscribers side. We implement a specific controller functionality in the broker process, which observes the delivery performance experienced by subscriber clients. In details, the broker works with temporal windows of *W* seconds: at the end of each temporal window, the broker estimates two performance measures for each subscriber:

- *Current hitrate H:* for evaluating the percentage of correctly forwarded messages, the broker waits for explicit notification from each subscriber. In other words, every *W* seconds each subscriber publishes to the broker the number of received messages *r*. In such a way, the broker can estimate the hitrate as the ratio between *r* and the number of forwarded messages *f*.
- *Average end-to-end latency L:* depending on the resources available on the clients, two options are available. If clients are all synchronized, the creation timestamp of each publication might be inserted in the publish messages so that subscribers might directly compute the end-to-end delay and notify to the broker its average value every *W* seconds. However, synchronization of clients might be complex to obtain in such a resource-constrained scenario. As a cheaper alternative, we propose to perform end-to-end delay estimation directly on the broker, by observing the number of application layer retransmissions performed. Indeed, the latency experienced by publish messages is directly correlated with the number of retransmissions performed by the broker towards the subscribers. The broker may therefore learn the relationship existing between latency and number of retransmissions and estimate the former from the latter. To verify this, we have used the simulation framework to transmit messages at different bit error rate values. Figure 4 shows the latency obtained by QoS 1 (from message publication to arrival of the PUBACK message at the broker) and QoS 2 messages (from message publication to arrival of the PUBREL message at the broker) depending on the number of retransmission observed. As one can see, there is a direct relationship between the number of retransmissions and the end-to-end latency: this happens because the application layer retransmission timer $T_{retry}$ is in the order of seconds and dominates over the MAC layer retransmission timer values of the 802.11b CSMA-CA access protocol by over three order of magnitude. Moreover, no matter what is the retransmission policy applied by the broker, the relationship between number of retransmissions and latency can be easily learned: as an example, Figure 5 shows two different retransmission policies, a linearly incremental retransmission timeout (in blue) and an exponential backoff where the retransmission timeout is doubled each time (in red).

### 4.1. Operational Modes

**Figure 6:**
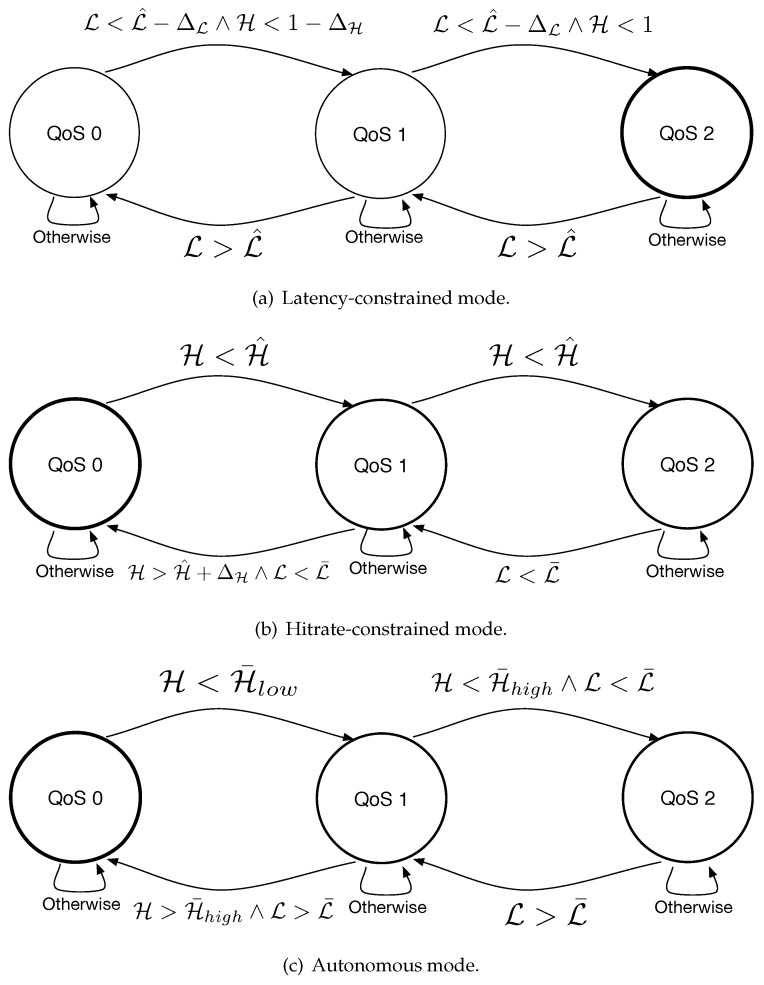
Finite state machines used to model the controller behavior for the three different operational modes: (**a**–**c**).

The controller is designed so as to work according to three different operational modes. Two modes accept delivery performance constraints directly from the subscribers and set their QoS levels in order to satisfy such constraints, possibly exploring other QoS levels when the performance requirements are largely matched. A third mode works autonomously, aiming at finding the best tradeoff between latency and hitrate for each subscriber. Each operational mode is described by a specific state machine, illustrated in Figure 6 and detailed below:

- *Latency-constrained mode:* in many real time and mission critical IoT applications [21], the subscribers have a tight constraint on the maximum delay that can be experienced. This operational mode allows the subscribers to express a latency constraints, $\hat{L}$, through a a specific control message transmitted after their connection to the broker. Upon the reception of such message, the controller will control the QoS level aiming at maximizing the hitrate achievable by each subscriber, subject to the latency constraint. With reference to Figure 6a, the controller initially sets all subscribers QoS level to 2, which corresponds to the maximum hitrate achievable. Then, at every decision window, the controller evaluates the hitrate H and latency L of each client as aforementioned. If the estimated latency L is greater than $\hat{L}$, the controller reduces the subscriber QoS to 1. At the next evaluation window, if the estimated latency is still above the constraints, the level is further reduced to QoS 0. In such a state, the controller also evaluates the current hitrate H of the subscribers: if the end to end latency satisfies the constraint by at least $\Delta_{L}$ seconds and H is below $1-\Delta_{H}$, the controller increases the QoS to 1 (we set $\Delta_{L}$ and $\Delta_{H}$ empirically). At the next evaluation window, the QoS can be still increased to QoS 2 if L still satisfies the constraint by at least $\Delta_{L}$ and the hitrate H is not at its maximum.
- *Hitrate-constrained mode:* to address delay-tolerant scenarios that require a specific amount of hitrate performance, the controller can also be set to work so as to minimize the end-to-end latency subject to a minimum hitrate constraint. The state diagram for this operation mode is illustrated in Figure 6b. In this case, the controller accepts the target hitrate $\hat{H}$ during the subscription phase and initially sets the QoS level of all subscribers to 0. Then, it proceeds at evaluating the current hitrate H and end-to-end latency L. If $H<\hat{H}$, the QoS level is increased to QoS 1. At the next round, if the target hitrate is still not reached, the QoS level is increased to QoS 2, where the hitrate reaches its maximum by definition. At each controller round, the QoS level may be decreased from QoS 2 to QoS 1 if the observed average end-to-end latency is below a specific threshold $\bar{L}$. Indeed, low end-to-end latency observed at QoS 2 implies good network conditions, therefore suggesting that a decrease in QoS would not impact the hitrate level too much, still reducing the latency and decreasing the number of control messages exchanged between the subscribers and the broker. After reaching QoS 1, the controller may still decrease the QoS to QoS 0 if the hitrate is at least $\Delta_{H}$ above the target hitrate and the observed latency is below $\bar{L}$, again suggesting good network conditions.
- *Autonomous mode:* Finally, the proposed QoS controller can also work in an autonomous mode, which seeks the best trade-off between latency and hitrate for each subscriber (Figure 6c). The controller works according to two hitrate thresholds, ${\bar{H}}_{low}$ and ${\bar{H}}_{high}$ and one latency threshold $\bar{L}$. At startup, the subscribers’ QoS is set to 0: if the observed hitrate is lower than ${\bar{H}}_{low}$, the controller increases the QoS to Qos 1. At this point, a new hitrate and latency estimation is performed: if the new hitrate has not exceeded ${\bar{H}}_{high}$ and the estimated latency is still below the threshold (suggesting good network conditions), the controller increases the QoS to QoS 2. Otherwise, if $H>{\bar{H}}_{high}$ and the latency is above the threshold, the controller sets the QoS back to QoS 0. Similarly, the QoS level is downgraded from QoS 2 to QoS 1 if $L>\bar{L}$. Note that depending on the network conditions and on the values given to the three thresholds used, there could exists loops between two adjacent QoS states. To avoid such issue, the controller tracks the QoS node of each subscriber and forces it to QoS 1 if loops are detected.

### 4.2. Practical Considerations

The proposed controller is designed to be implemented on board the MQTT broker behind the MQTT-SN gateway. Here, we present some proposals for a seamless integration of the controller functionalities in the latest MQTT standard on the broker side.

**Figure 7:**
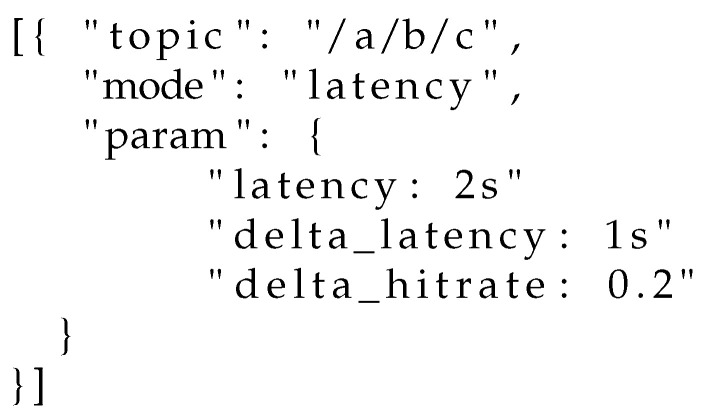
Activation of the QoS controller in latency-constrained mode.

**Figure 8:**
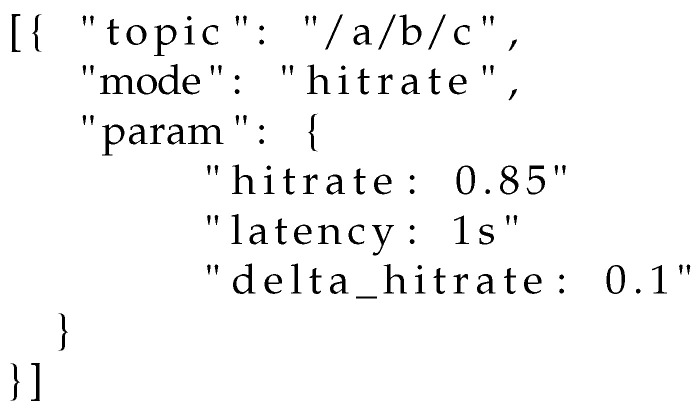
Activation of the QoS controller in hitrate-constrained mode.

**Figure 9:**
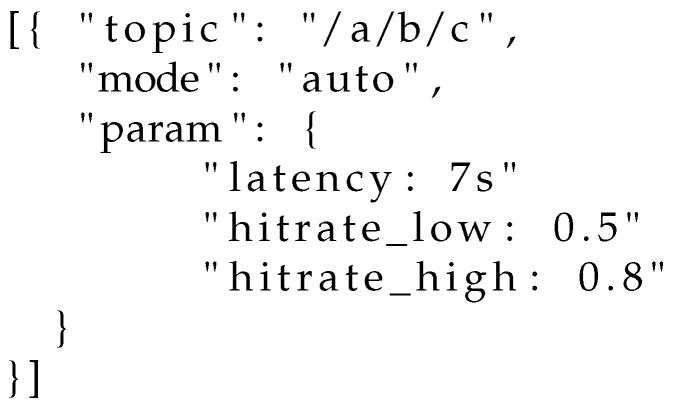
Activation of the QoS controller in autonomous mode.

- *Controller activation:* the QoS controller may be activated by any subscriber at will. Due to the limited options available in the current subscribe message packet header and payload, which must follow a very specific syntax according to the MQTT standard, we propose the following strategy. Upon subscription on any topic, a client may send to the gateway/broker a specific control message on the special topic $QoSControl/Activate, passing as a payload a JSON file containing (i) the topic over which the QoS controller should work, (ii) the operational mode and (iii) the operational parameters (constraints and thresholds). Examples are illustrated in Figure 7, Figure 8 and Figure 9.
- *Feedback communication:* Regardless of the operational mode selected by subscribers, the controller needs to be aware of (i) the current hitrate and (ii) the average end-to-end latency experienced by nodes in the current evaluation window. While for the latter metric the broker can perform an estimation based on the number of retransmissions performed, for what concerns the current hitrate each client needs to explicitly communicate the number of received messages in the previous evaluation window. Such an exchange may happen on a special control topic, such as, e.g., $HITRATE/topic. In such a way, the broker can be aware of the hitrate each client is observing on the topic topic.
- *Controller deactivation:* A client has two choices for deactivating the controller: (i) unsubscribing from the topic over which the control is active, and resubscribing without activation or (ii) transmitting an explicit message on the special topic $QoSControl/Dectivate, passing as payload a JSON file where the value of mode is equal to off.

We highlight that, to provide the maximum degree of flexibility, the proposed controller works independently on each subscriber requesting its activation. Indeed, each subscriber connected to the broker may: (i) not use the controller and rely on the standard QoS levels, (ii) activate the controller specifying its own latency or hitrate constraints or (iii) use the controller in autonomous mode.

## 5. Experimental Evaluation

We performed several simulation experiments to test the performance of the proposed QoS controller, using the same network configuration illustrated in Figure 2. We present the obtained results as a function of the Packet Error Rate (PER) experienced by subscribers, i.e., the fraction of packets lost by subscribers. Note that the PER is determined by both the channel BER as well as the level of interference traffic. To easily control the number of lost packets at the application layer, we programmed the simulator setting an ideal channel (with no losses and no interference traffic) and we forced the UDP layer at each subscriber to drop a specific number of packets, therefore triggering retransmissions at the application layer. We find this approach more practical than carefully setting the lower layers parameters (BER and interference traffic) to reach a specific PER. Moreover, such an approach allows us to shadow the complexity of the lower layers of the protocol stack, allowing us to obtain results at the application layer, which are valid regardless of the specific choices performed at the lower layers (e.g., MAC + PHY configuration, level of interference, signal quality and BER, etc.). Therefore, in each simulation performed, we set the average PER $\bar{p}$ of the network, assigning to each subscriber a specific PER value drawn from a normal distribution with mean $\mu =\bar{p}$ and standard deviation σ.

During all tests, the simulation is performed by setting one publisher to send periodically one 1400-byte message every second, for a total duration of 5 min. For the first 120 s of the execution, the controller is left shut down to show the behavior without its adjustment. After 120 s the controller starts its execution by checking the metrics of the previous messages and adjusting the QoS of the subscribers, if needed. Finally, to avoid continuous change of the QoS of a single subscriber, the controller can not change the QoS of the same subscriber in two consecutive intervals.

### 5.1. Runtime Behavior

**Figure 10:**
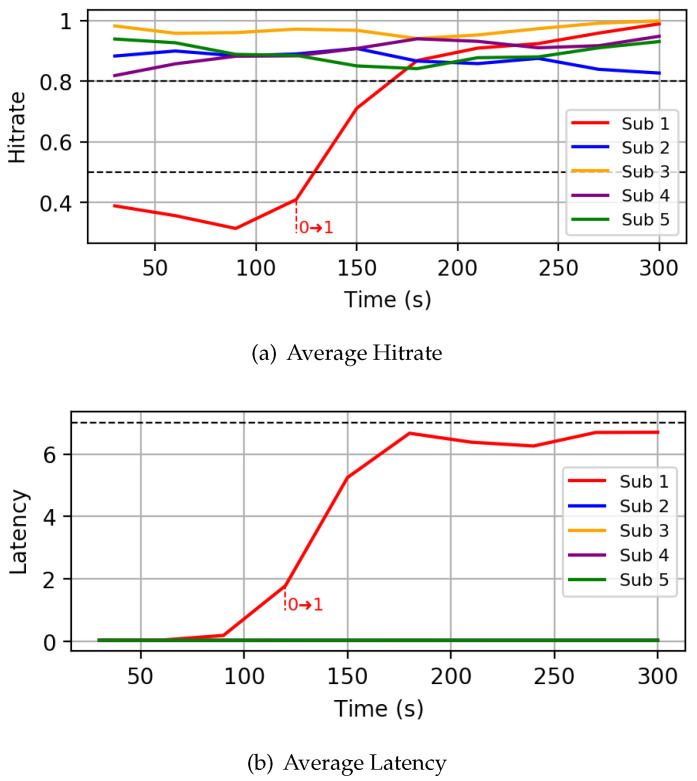
Autonomous mode scenario. The black dotted lines indicate the thresholds used during the simulation.

**Figure 11:**
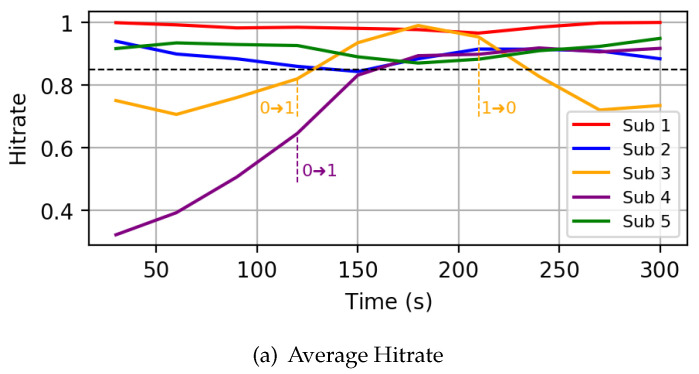

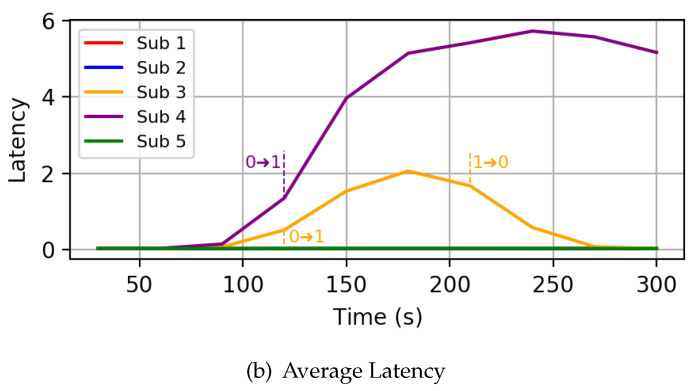
Hitrate-constrained mode scenario.

**Figure 12:**
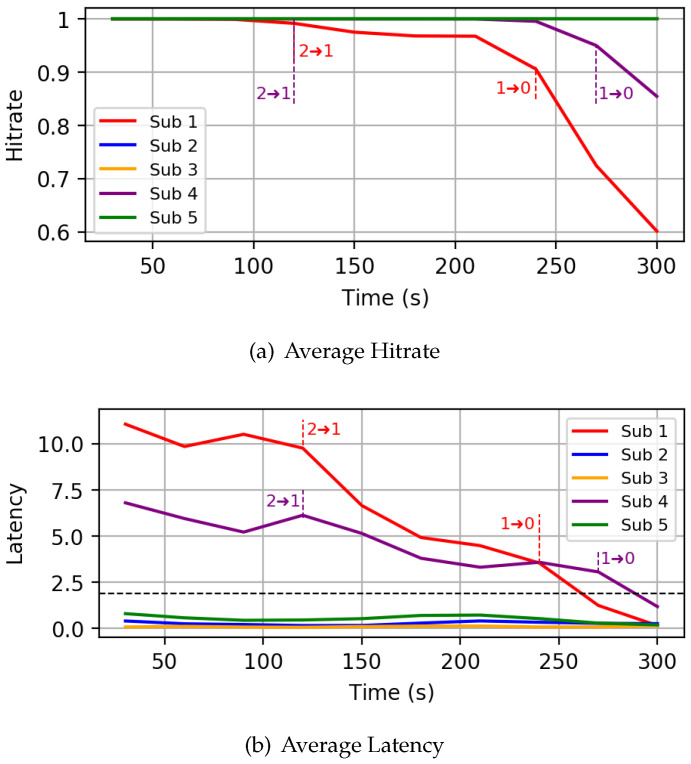
Latency-constrained mode scenario.

- *Autonomous mode:* As a first experiment, we run the simulation framework with five subscribers, activating the controller in autonomous mode after 120 s. The following parameters have been used to setup the controller: ${\bar{H}}_{low}$ = 0.5, ${\bar{H}}_{high}$ = 0.8 and $\bar{L}$ = 7s. We focus on a specific realization where one of the subscriber (Sub 1) is characterized by a very high PER of 0.6, while the others have a PER of around 0.1. Figure 10 shows the hitrate and latency experienced by the subscribers throughout the simulation. At the beginning, all subscribers start at QoS 0, and therefore experience very low latency. As one can see, when the controller is activated at 120 s, the QoS of Sub 1 is increased from QoS 0 to QoS 1, causing an increase in hitrate and a corresponding increase in latency. No further changes are performed by the controller, according to the state machine illustrated in Figure 6c, since the reached hitrate for Sub 1 is already higher than ${\bar{H}}_{high}$ and its new latency is below $\bar{L}$. All the other nodes require no changes, since their hitrate is already higher than ${\bar{H}}_{high}$.
- *Hitrate-constrained mode:* As a second experiment, we run the same network simulation as before, activating the controller in hitrate-constrained mode. The following parameters have been used: the target hitrate H has been set to 0.85, the hitrate margin $\Delta_{H}$ to 0.1 and the latency threshold $\bar{L}$ to 5 s. In this case, two nodes (Sub 3 and Sub 4) are characterized by a PER greater than 0.2. Figure 11a illustrates the behavior of the hitrate experienced by nodes in the simulation, while Figure 11b does the same for the end-to-end latency. As one can see, after the controller is started, the QoS of nodes 3 and 4 is increased to QoS 1, greatly improving the hitrate. After some iterations though, the QoS of Sub 3 is reduced to QoS 0 as the hitrate is above the safe margin $\Delta_{H}$, while the latency is below the threshold. This produces a new hitrate which violates the input constraint. Such a behavior would produce a loop between the two states, with the controller continuously increasing and decreasing the QoS level. To avoid such issue, we implemented a QoS loop detection mechanism, which forces the higher QoS level in case a loop is detected. In this specific case, the controller forces QoS 1 on Sub 3.
- *Latency-constrained mode:* Finally, we also report the runtime behavior for a scenario where the controller was run in latency-constrained mode, setting the target latency $\bar{L}$ to 2 s and the latency and hitrate margins ($\Delta_{L}$ and $\Delta_{H}$) to 1 s and 0.2, respectively. In this simulation, nodes 1 and 4 are characterized by a PER above 0.35, while the other subscribers have a PER below 0.1. All nodes start with QoS 2 by default setting. As one can see from Figure 12b, subscribers 1 and 4 are initially characterized by latencies higher than the target one: therefore, upon its activation, the controller reduces their QoS from QoS 2 to QoS 1 after 120 s. This has the effect of reducing the latency on the one hand, but on the other hand also decreases the hitrate of the two nodes (Figure 12a). Note that sub 1 (red line) has a faster decrease of hitrate compared to node 4. Anyway, for both subscribers, since the newly estimated latency is above the constraint of 2 s, the controller sets their QoS to 0, further decreasing their hitrate.

### 5.2. Performance Comparison

Finally, we performed several experiments to compare the performance of the proposed controller with the ones obtained by (i) the legacy MQTT-SN QoS levels and (ii) the method proposed in [20] (indicated as Rocha2019). In order to do this, we set up a network topology with 20 subscribers: again, the PER of each subscriber is set randomly, according to a normal distribution with mean $\bar{p}$ and standard deviation 0.1. We performed several experiments, each time increasing the value of the average PER $\bar{p}$. For each scenario, we extracted from the simulation the mean hitrate and latency observed by each subscriber over the entire simulation length, either forcing the QoS to a fixed level or using the controller in different operational modes. The input parameters of the different operational modes are the same that have been used in the runtime behavior experiments.

To perform a fair comparison with the method in [20], we performed the following assumptions: (i) the QoS controller is applied on the subscribers, (ii) the subscribers all request QoS 1 and (iii) the network latency estimation is performed end-to-end, exactly as in our case. The latency thresholds for normal network conditions (when the controller in [20] leaves the QoS level untouched) are set to 1 s and 3 s. Note that if the estimated latency is below the lower thresholds or above the higher, the method in [20] will force the subscribers to QoS 0 level.

**Figure 13:**
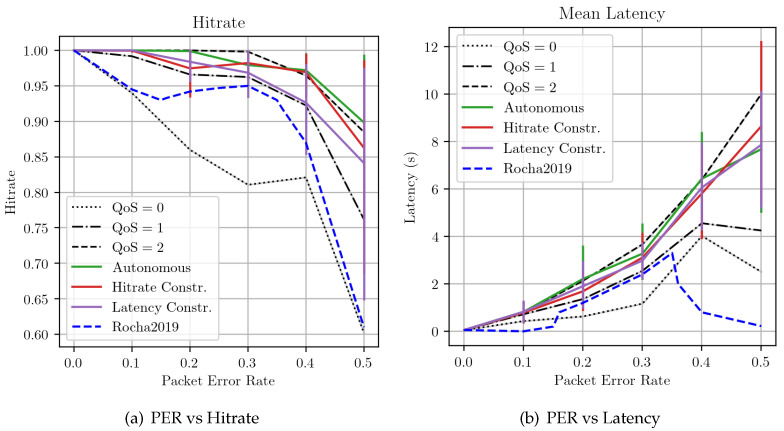
Average hitrate and latency for different scenarios with or without the proposed QoS controller. The method indicated as Rocha2020 refers to the work in [20].

At the end of each simulation, we aggregate the results obtained using their average and standard deviation. The results are illustrated in Figure 13. As one can see, such results again highlight the tradeoff present between hitrate and latency. At very low PER values, all configurations tested result in good performance. However, as the PER increases, it is clearly visible how the case where all subscribers have been forced to QoS 0 departs from all other configurations, with a rapidly decreasing hitrate. Conversely, the proposed QoS controller has performance that is between the ones showed by the QoS 1 and QoS 2 fixed scenarios, both in terms of hitrate and latency, with the plus of respecting the delivery performance requested by clients. Finally, the method in [20] works as expected, with the average hitrate and latency behaving as QoS 0 when the estimated latency is outside the interval corresponding to normal network conditions and with performance closer to QoS 1 (the original level requested by the subscribers) otherwise.

## 6. Conclusions

This work has presented the design and implementation of a QoS controller for MQTT-SN applications built on lossy wireless networks. In such cases, using the fixed QoS levels provided by the MQTT/MQTT-SN standards may be not straighforward, since the actual QoS experienced by nodes depends on the underlying network characteristics, which are generally unknown to clients. Therefore, we propose a controller that runs on the broker and accepts as input from the subscribers explicit performance delivery constraints in terms of average latency or hitrate. The controller is able to work in different operational modes to optimize the preferred metric, as well as in an autonomous mode, which seeks the best tradeoff between the two. We implemented the controller in a simulation environment based on the ns-3 network simulator framework and tested its behavior in a variety of cases. We performed several experiments to validate the simulation framework, show the live behavior of the proposed controller and summarize its performance. Future research directions will target the real-life implementation and testing of the proposed controller in one of the available MQTT/MQTT-SN brokers.

## References

[1] Cesana M., Redondi A.E. (2017). Iot communication technologies for smart cities. Designing, Developing, and Facilitating Smart Cities.

[2] Banks A., Briggs E., Borgendale K., Gupta R. (2019). MQTT Version 5.0.

[3] Stanford-Clark A., Truong H.L. (2013). MQTT-SN Version 1.2.

[4] Capone A., Fratta L., Martignon F. (2004). Bandwidth estimation schemes for TCP over wireless networks. IEEE Trans. Mob. Comput..

[5] Dilek S., Irgan K., Guzel M., Ozdemir S., Baydere S., Charnsripinyo C. (2022). QoS-aware IoT networks and protocols: A comprehensive survey. Int. J. Commun. Syst..

[6] Lo N., Niang I. A Survey on QoS-based communication protocols for IoT systems. Proceedings of the 3rd International Conference on Networking, Information Systems & Security.

[7] White G., Nallur V., Clarke S. (2017). Quality of service approaches in IoT: A systematic mapping. J. Syst. Softw..

[8] Palmese F., Longo E., Redondi A.E., Cesana M. CoAP vs. MQTT-SN: Comparison and Performance Evaluation in Publish-Subscribe Environments. Proceedings of the 2021 IEEE 7th World Forum on Internet of Things (WF-IoT).

[9] Saint-Andre P. (2011). Extensible Messaging and Presence Protocol (XMPP): Core.

[10] Kramer J. (2009). Advanced message queuing protocol (AMQP). Linux J..

[11] Betzler A., Gomez C., Demirkol I., Paradells J. (2016). CoAP congestion control for the internet of things. IEEE Commun. Mag..

[12] Betzler A., Gomez C., Demirkol I., Paradells J. (2015). CoCoA+: An advanced congestion control mechanism for CoAP. Ad Hoc Netw..

[13] Ludovici A., Garcia E., Gimeno X., Augé A.C. Adding QoS support for timeliness to the observe extension of CoAP. Proceedings of the 2012 IEEE 8th International Conference on Wireless and Mobile Computing, Networking and Communications (WiMob).

[14] Jo H.C., Jin H.W. Adaptive periodic communication over MQTT for large-scale cyber-physical systems. Proceedings of the 2015 IEEE 3rd International Conference on Cyber-Physical Systems, Networks, and Applications.

[15] Sadeq A.S., Hassan R., Al-rawi S.S., Jubair A.M., Aman A.H.M. A qos approach for Internet of Things (Iot) environment using mqtt protocol. Proceedings of the 2019 International Conference on Cybersecurity (ICoCSec).

[16] Vatcharatiansakul N., Tuwanut P. (2016). Adaptive Publish Time and QoS Level over MQTT Protocol. IEICE Proc. Ser..

[17] Zhang H., Zhang H., Wang Z., Zhou Z., Wang Q., Xu G., Yang J., Gan Z. (2022). Delay-reliability-aware protocol adaption and quality of service guarantee for message queuing telemetry transport-empowered electric Internet of things. Int. J. Distrib. Sens. Netw..

[18] Rausch T., Nastic S., Dustdar S. Emma: Distributed qos-aware mqtt middleware for edge computing applications. Proceedings of the 2018 IEEE International Conference on Cloud Engineering (IC2E).

[19] Govindan K., Azad A.P. End-to-end service assurance in IoT MQTT-SN. Proceedings of the 2015 12th Annual IEEE Consumer Communications and Networking Conference (CCNC).

[20] Rocha H.d., Monteiro T.L., Pellenz M.E., Penna M.C., Alves Junior J. (2019). An MQTT-SN-based QoS dynamic adaptation method for wireless sensor networks. Proceedings of the International Conference on Advanced Information Networking and Applications.

[21] Guo X., Han S., Hu X.S., Jiao X., Jin Y., Kong F., Lemmon M. Towards Scalable, Secure, and Smart Mission-Critical IoT Systems: Review and Vision:(Special Session Paper). Proceedings of the 2021 International Conference on Embedded Software (EMSOFT).

